# The Boiling Phenomena and their Proper Identification and Discrimination Methodology

**DOI:** 10.1038/s41598-020-65342-0

**Published:** 2020-05-20

**Authors:** A. R. Pati, B. Swain, S. S. Mohapatra

**Affiliations:** 1 Department of Chemical Engineering, Spray Boiling Heat Transfer Laboratory, National Institute of Technology Rourkela, Rourkela, 769008 India; 2 Department of Metallurgical and Materials Engineering, National Institute of Technology Rourkela, Rourkela, 769008 India

**Keywords:** Chemical engineering, Chemical engineering, Fluid dynamics, Fluid dynamics

## Abstract

The proper identification of the existence of the boiling phenomena in a process from the trend of the Nukiyama pool-boiling curve (*Q* versus *∆T*) is not appropriate as it does not always reveal right information. All the heating and cooling around the boiling point mimic the boiling behavior; however, these are not always actually a part of the boiling process. Therefore, the proper identification and discrimination among boiling methodologies need to be revealed as the information on the discussed issues are not available in the open literature. Hence, an attempt has been made to develop a condition describing the existence of boiling behavior in heating or quenching process and protocol to identify various boiling regimes. In the current work, the developed conditions (*1/ St*) are validated with various type of boiling processes and the protocol (i.e. based on the slope of *h* versus *∆T* curve rather than boiling curve) is also valid for the identification of proper boiling regime.

## Introduction

Boiling is a solid-liquid interfacial behavior which is observed during various types of quenching such as cooling by pool of liquid, spray, air atomized spray, water jet and siphons^1,2^. The various boiling behaviours such as nucleate, transition and film are controlled and defined by evaporation rate and associated heat transfer mechanism^3^. The literatures reveal that the boiling behaviors are generally identified from the nature of the variation of heat flux (*Q*) with wall superheat^4^ (*ΔT*). The discussed identification methodology follows the technique proposed by Nukiyama^5^ in pool boiling investigation.

In open literature, it is recommended that the boiling behaviour is identified from the nature of the *Q* versus *ΔT* diagram which is called as boiling diagram^6,7^. Furthermore, the literature indicates that the amount and the nature of energies extracted in case of very slow boiling process are almost similar to the heat removal rate achieved for intense forced convection cooling by air^8^. Therefore, without the existence of the boiling behaviour in many cooling processes, a curve, which is qualitatively similar to the nature of the boiling diagram generates. As a result, the conclusion obtained from the comparison made with the standard boiling diagram^5^ cannot assure the existence of boiling behaviour in the process. The proper identification and discrimination need condition defining the aforesaid. The literature does not indicate the availability of any conditions indicating boiling behaviour and therefore, in the current work, an attempt has been made to develop conditions depicting the existence or non-existence of boiling behavior in the process.

In case of quenching by conventional spray and air atomized spray, the heat transfer behaviour is characterized or defined from the nature of boiling predicted by boiling diagram. For the onset of boiling at the solid-liquid interface, the droplet or the thin liquid film has to elapse sufficient time for the nucleation and their growth^9–11^. Furthermore, the discussed phenomenon also depends on thermo-physical properties of the fluid defining the heat transfer coefficient (*h*). Therefore, the occurrence of boiling at the solid-liquid interface is controlled by residence time of the coolant on the quenching surface and thermo-physical properties of the fluid^3,12^. Nguyen *et al*.^13^ have considered oscillate boiling technique to overcome the boiling crisis phenomena occurred in microheaters. They have attained stable oscillate boiling and high heat transfer rate by operating at high power. A self-propulsion and self-centred technique using a negative feedback principle was introduced by Dodd et al. to control the droplet motion along a user defined path by means of the Leidenfrost effect. Herringbone and ratchet design was used by the above researchers to regulate the droplets^14^. The similar type of explorations were carried out by many researchers^15–17^.

For the fast quenching operations, the existence of the boiling in the process is mandatory and as a result, it is essential for the identification of condition predicting the boiling behaviour. Hence, in the current work, an attempt has been made to develop a protocol for the identification of the existence of boiling behaviour in various thermal processes. In case of boiling along with the above-discussed parameter, temperature of liquid or hot substrate is considered as the another controlling parameter for the onset of boiling behavior^18^. For the initiation of boiling, the local temperature of the liquid must reach up to the boiling temperature. Therefore, with the help of proper energy balance, appropriate conditions are determined and also validated for different quenching processes such as air atomized spray, spray cooling and forced convection cooling.

## Discussion

### Condition 1

**Figure 1:**
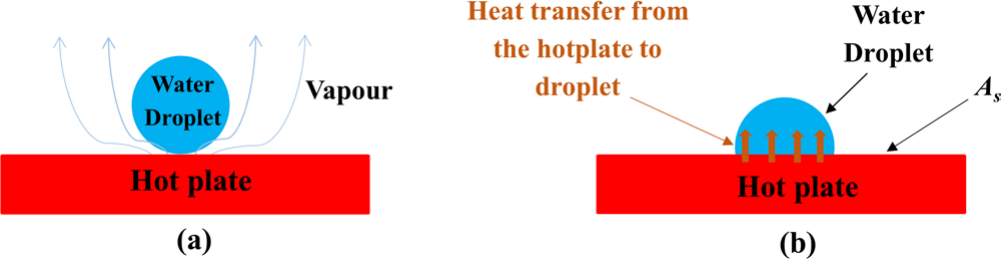
Schematic diagram of droplet (**a**) at t = 1 ms (**b**) at t = 20 ms.

For the initiation of boiling (Fig. 1), the sensible heat part of the impinging droplet must be less than the heat transferred from hot plate to the water droplet by various thermal mechanisms.

1 $$\frac{m\,{C}_{p}}{t}({T}_{b}-{T}_{f}) < h\,{A}_{s}\,({T}_{s}-{T}_{f})$$

2 $${\rm{Again}},\frac{m}{t}=v\,\rho \,{A}_{c}$$

Substituting the value of $\frac{m}{t}$ in Eq. 1,

3 $$v\,\rho \,{A}_{c}\,{C}_{p}({T}_{b}-{T}_{f}) < h\,{A}_{s}\,({T}_{s}-{T}_{f})$$

Rearranging Eq. 3,

4 $$\frac{v\,\rho \,{C}_{p}}{h\,} < \frac{({T}_{s}-{T}_{f}){A}_{s}}{({T}_{b}-{T}_{f}){A}_{c}}$$

5 $$\frac{\dot{m}\,{C}_{p}}{h\,{A}_{s}} < \frac{({T}_{s}-{T}_{f})}{({T}_{b}-{T}_{f})}$$

where $m=$ Mass of the liquid (kg), ${C}_{p}$ = Specific heat of the liquid (J/kg °C), ${T}_{s}=$ Initial temperature of the substrate (°C), ${T}_{b}=$ Boiling temperature of the liquid (°C), ${T}_{f}=$ Final temperature of the substrate (°C), $h=$ Heat transfer coefficient (W/m^2^ °C), ${A}_{s}=$ Surface area of the plate (m^2^), $t=$ Residence time of the liquid on the hot surface (s), *ν* = Velocity of the droplet (m/s), *ρ* = Density of the droplet (kg/m^3^), ${A}_{c}=$ Surface area of the droplet (m^2^) and $\dot{m}=$ mass flow rate (kg/s) $,m/t$.

If the considered fluid is water at normal temperature and the substrate temperature is at ${T}_{s}$ (°C), then the equation further modifies to following form.

6 $$\frac{\dot{m}\,{C}_{p}}{h\,{A}_{s}\,} < \frac{{T}_{s}-25}{75}$$

7 $$\frac{1}{St} < \frac{{T}_{s}-25}{75}$$

Mass flow rate is determined from spray impingement density of the spray^19^, which is calculated by using Eq. 8.

8 $${I}_{d}=\frac{4\,{M}_{w}}{\pi \,{d}_{t}^{2}\,\varDelta t}$$

The residence time for each droplet can be calculated by using the formula^19^ given in Eq. 9.

9 $$t=\left(\frac{{m}_{d}}{{I}_{d}\,{A}_{c}}\right)+\left(\frac{r}{{v}_{d}}\right)$$

To initiate any type of boiling, the process must obey the condition given in Eq. 6.

Similarly, in case of nucleate boiling regime,

10 $$\dot{m}\,{C}_{p}({T}_{b}-{T}_{f})+\sigma \,\dot{m}\,\lambda  < {h}_{1}\,{A}_{s}\,({T}_{s}-{T}_{f})$$

In transition boiling regime,

11 $$\dot{m}\,{C}_{p}\,({T}_{b}-{T}_{f})+\sigma \,\dot{m}\,\lambda  < ({x}_{1}{h}_{1}+{x}_{2}{h}_{2}){A}_{s}({T}_{s}-{T}_{f})+\sigma \,A({T}_{s}^{4}-{T}_{f}^{4})$$

In film boiling regime,

12 $$\dot{m}\,{C}_{p}({T}_{b}-{T}_{f})+\sigma \,\dot{m}\,\lambda  < {h}_{2}\,{A}_{s}({T}_{s}-{T}_{f})+\sigma A({T}_{s}^{4}-{T}_{f}^{4})$$

where *St* = Stanton number (= $\frac{h}{\rho \,u\,{C}_{p}}or\frac{h\,{A}_{s}}{\,\dot{m}\,{C}_{p}}$), ${I}_{d}=$ Impingement density or mass flux (kg/m^2^s), *M*_*w*_ = Mass of water collected for measuring the mass flux (kg), *d*_*t*_ *=* Diameter of the tube (m), *Δt* = Time interval (*s*), *m*_*d*_ = Mass of water droplet (kg), *r* = Distance travelled by the droplet or liquid film on the hot plate (m), *v*_*d*_ = Velocity of the droplet or liquid film on the hot plate (m/s), *σ* = Distribution coefficient, ${h}_{1}$ = heat transfer coefficient for liquid film (W/m^2^ °C), ${h}_{2}$ = heat transfer coefficient in case heat transfer occurs from solid to liquid through the vapour blanket (W/m^2^ °C), *x*_*1*_ and *x*_*2*_ = Fraction of vapour and liquid and *λ* = Latent heat of vaporization (J/kg).

### Condition 2

13 $$\begin{array}{c}{\rm{As}}\,Bi=\frac{h\,{L}_{c}}{k}\\ {\rm{Again}},\dot{m}\,\lambda =h\,A\Delta T\end{array}$$

Substituting the value of *h* in Eq. 13,

14 $$Bi=\frac{\dot{m}\,\lambda \,{L}_{c}}{A\,\Delta T\,k}$$

For sphere, the characteristic length (${L}_{c}$) is $R/3$

Equation 14 becomes

15 $$Bi=\left(\frac{\dot{m}\,\lambda }{A\,\Delta T\,k\,}\right)\left(\frac{R}{3}\right)$$

where ${L}_{c}=$ Characteristic length (m), *ΔT* = wall superheat (°C), $k$ = Thermal conductivity of the solid (W/m °C), *R* = Radius of the droplet (m) and $Bi=$ Biot number

**Figure 2:**
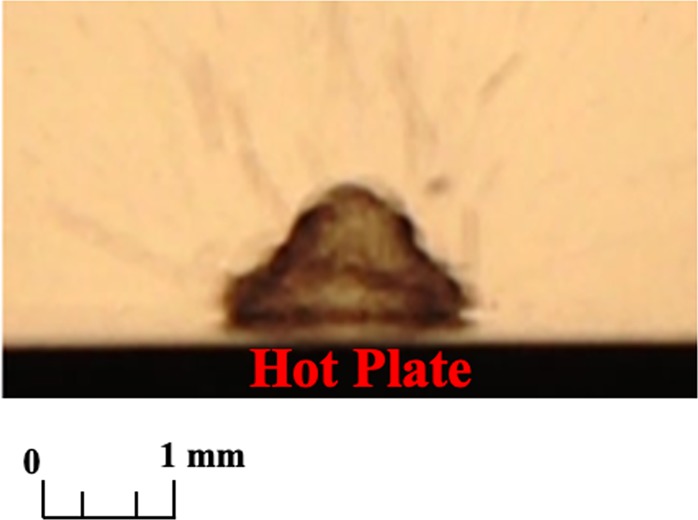
The dropwise evaporation in case of *Bi* < *0.1*.

**Figure 3:**
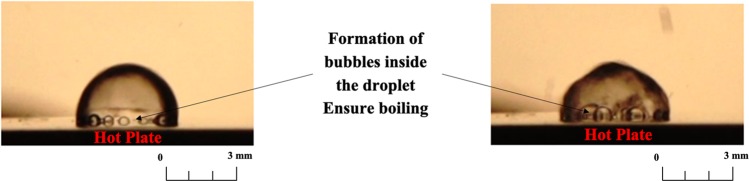
The dropwise evaporation in case of *Bi* > *1*.

If $\,Bi$ is less than 0.1, the heat transfer follows lumped capacitance theory due to very low film resistance. In this case, the existence of the discussed characteristic in the evaporating droplet does not allow the boiling behavior to develop. For the experimental verification (Fig. 2), the parameters defining *Bi <0.1* in a process were used. The images captured by high speed motion analyzer (SONY, RX100-V) clearly ascertain the absence of boiling phenomena at the solid-liquid interface. Furthermore, when *Bi* > *1*, the experimental result presented in Fig. 3 assures the existence of boiling phenomenon in the process.

**Figure 4:**
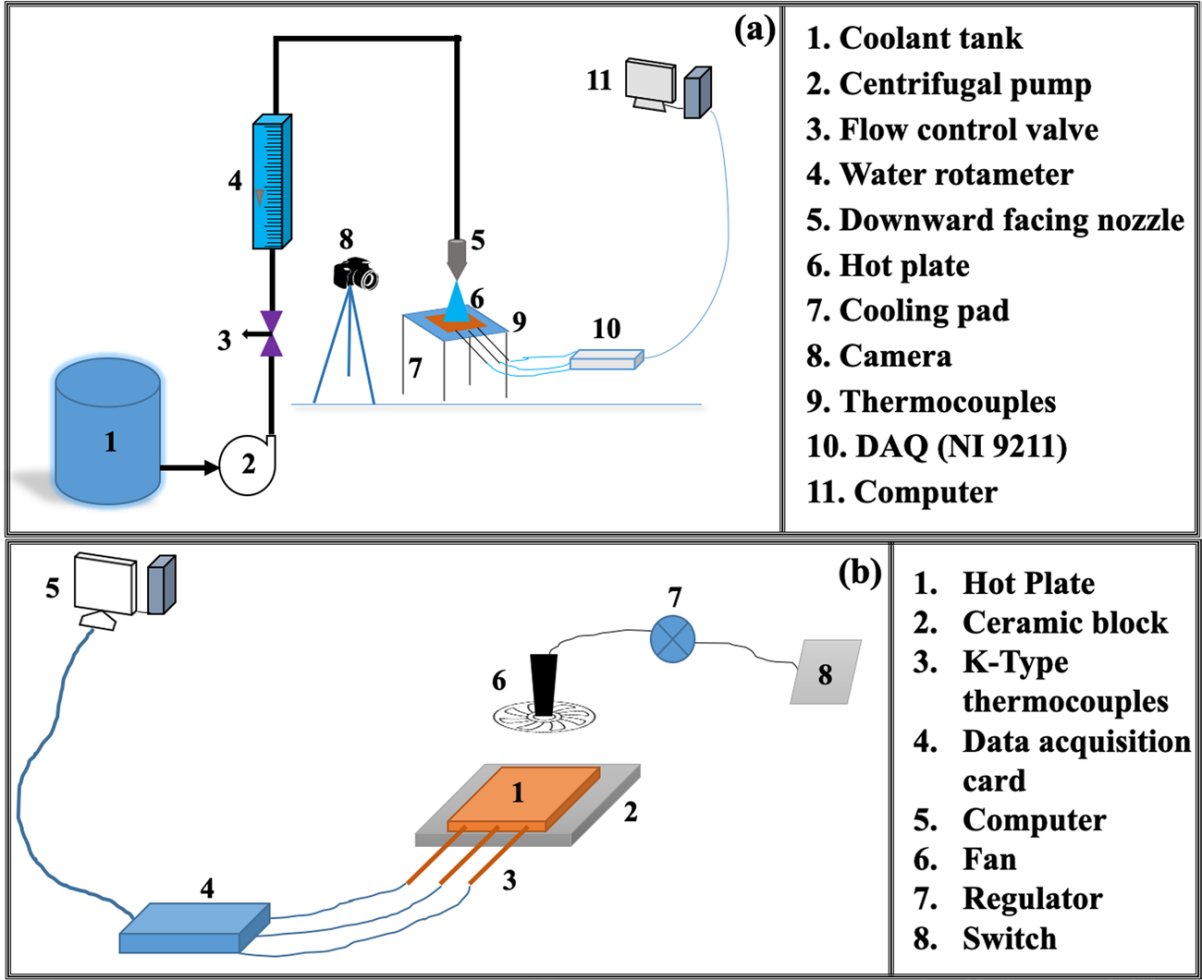
Schematic diagram showing the experimental setup for (**a**) Spray cooling (**b**) forced convection cooling by air.

To identify the nature of heat flux variation with wall superheat, experiments were conducted for three different mass fluxes in case of conventional spray cooling and forced convection cooling by air are shown in Fig. 4a,b, respectively. In addition to the above, the dropwise evaporative experiments and the air atomized experiments were also performed as mapped in the literature^12,20^. Experiments were conducted at various optimum level of parameters such as nozzle height and mass flux^21^. To avoid the oxide layer effect, experiments were conducted on low carbon steel plate i.e. AISI 304. The post experiments analysis which include determination of surface heat flux and temperature have been performed by using an inverse heat conduction algorithm developed by Bushby and Trujilo^22,23^. To avoid the error induced various parameters in the inverse calculation, all the recommended precautions were implemented^24^.

#### Validation of condition 1

**Figure 5:**
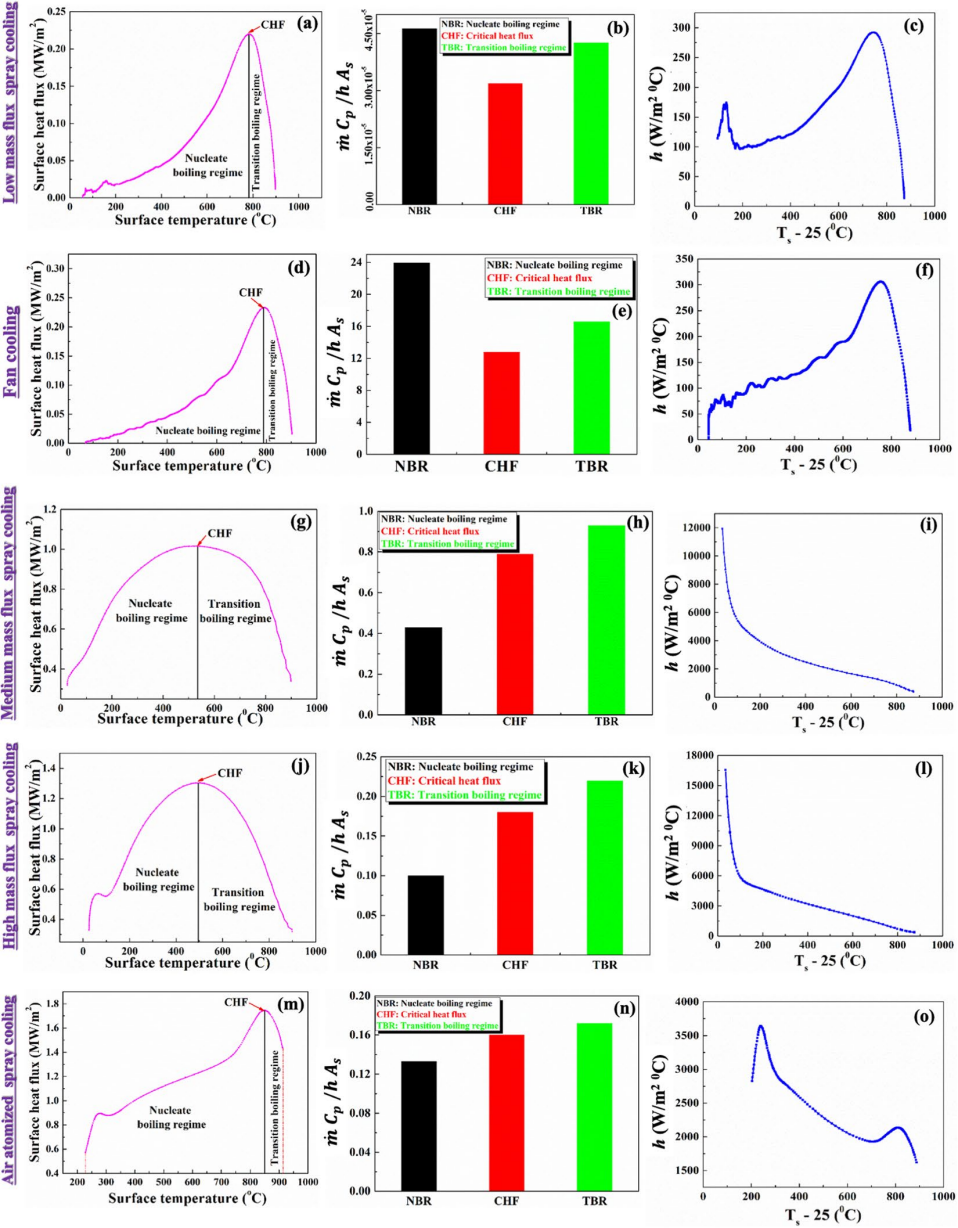
Variation of surface heat flux with surface temperature for (**a,d,g,j,m**); Validation with Eq. 6 for (**b,e,h,k,n**); Variation of heat transfer coefficient with wall superheat for (**c,f,i,l,o**).

**Figure 6:**
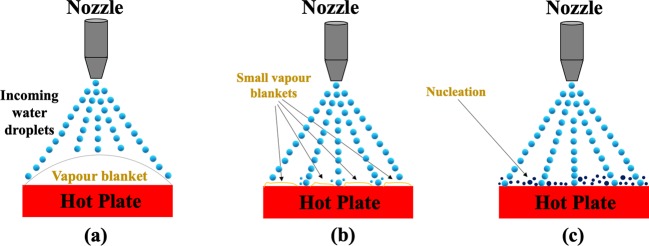
Schematic diagram of (**a**) Film boiling, (**b**) Transition boiling (**c**) Nucleate boiling. The dark blue circles represent the nucleation process.

The validity of the developed condition 1 is initially checked for very low mass flux (${I}_{d}$= 0.027 kg/m^2^ s) spray cooling (Fig. 5(a)). For nucleate and transition boiling regimes and at the critical heat flux point, the developed condition is checked and it is corroborated (Fig. 5(b)**)** that the developed condition successfully predicts the boiling behaviour. The observed value is much lower than the set condition. Therefore, it is expected that significant amount of evaporation occurs at the solid-liquid interface. The schematic diagrams of the various boiling regimes are also illustrated in Fig. 6.

In few cases, it is observed that the cooling curve qualitatively depicts exactly same nature as the boiling curve; however, the outcome is not the result of the boiling behavior. Therefore, to check the developed condition in case of the discussed process, the forced convection cooling experiments were performed by using a fan with speed of 4000 rpm. During the experimentation, the time-temperature histories were recorded and then by using INTEMP software and the surface heat flux is determined at various wall superheats. The variation of surface heat flux with wall superheat is presented in Fig. 5(d) and it can be said that the presented Fig. 5(d) mimics the nature of boiling diagram. However, with the help of Eq. 6, it is observed that the condition is not valid for all the considered regimes and at critical heat flux point. Therefore, it can be concluded that nature of the *Q* versus *ΔT* curve is not the appropriate methodology to decide the boiling behavior.

After confirmation of existence of boiling behavior in different processes from the information of *Q* versus *ΔT* diagram by using the developed condition, the proper discrimination of nucleate (Fig. 6(c)**)** and transition boiling regime (Fig. 6(b)**)** is essential. From the nature of *Q* versus *ΔT* diagram, the aforesaid cannot be decided. According to qualitative analysis performed in the literature, transition boiling regime slope is generally lower than the nucleate boiling due to the achievement of high heat removal rate in nucleate boiling. In case of high mass flux spray and air-atomized spray, boiling diagrams of different nature are obtained. The boiling diagram of high mass flux spray cooling depicts almost same slope in nucleate and transition boiling regimes and whereas air-atomized spray shows higher slope in transition boiling regime. Therefore, from the above discussion, it cannot be predicted about the boiling regimes. For this, the identification of nucleate and transition boiling regimes are confirmed from the variation of heat transfer coefficient with the wall superheat. The value of *h* and the slope between *h* and wall superheat are higher in nucleate boiling regime (Fig. 5(i,l)). Therefore, the discussed approach can be considered as one protocol for identification of various boiling regimes.

If the *Q* versus *ΔT* variation exhibits the nature presented in Fig. 5(m), then it is very difficult to identify the existence of boiling and discrimination of various regimes. Thus, proper identification requires condition simulating the boiling behavior and regime, which is presented in Eq. 6. From the condition, the existence of boiling in the process is assured **(**Fig. 5(n)**)** and various regimes are identified with the help of the proposed protocol.

Based on the developed model and analysis of the experimental results, the followings are the conclusions:

1. For the existence of boiling in a process, the inverse Stanton number (*St*) must be lower than ($\frac{{T}_{s}-25}{75}$). The low mass flux, high mass flux and air atomized spray validate the developed condition and whereas the fan cooling does not.
2. The nucleate and transition boiling regimes identification are recommended to predict from the slope of *h* with wall superheat rather than from boiling diagram. Experimental results of three different cases confirm the above-mentioned recommendation.
3. Without the existence of boiling, the curve which is similar to the boiling curve proposed by Nukiyama can also be observed.

### Data processing

#### Calculation of surface heat flux and surface temperature

**Figure 7:**
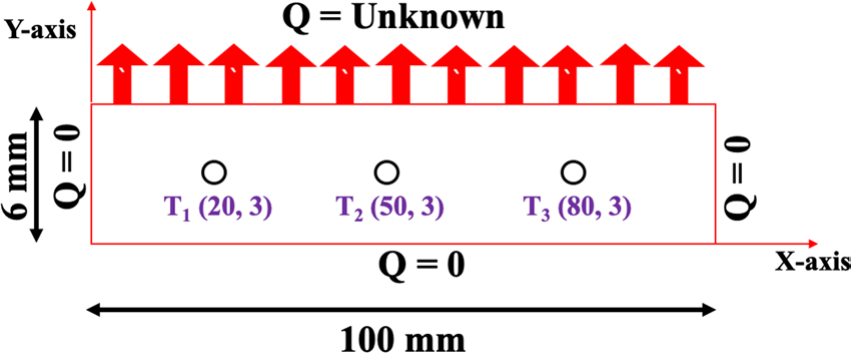
Computational domain of the steel plate (i.e T_1_(20,3) – Thermocouple number (X coordinate, Y coordinate)).

The surface heat flux and surface temperature calculations in the current investigation deal with an inverse heat conduction software known as “INTEMP”. A 2D heat transfer model was considered to estimate the heat transfer behavior inside the hot plate. The studied steel plate was discretized into 3340 quadratic elements and each element consists of four nodes. Figure 7 shows the computational domain of the steel plate. The assumptions, input and the algorithm for the above calculation are given below.

##### Assumptions and inputs

1. The top surface was divided into three heat flux regimes.
2. Except the impinging surface, all the other surfaces should be kept adiabatic during the experiments.
3. The material properties of the steel plate are assumed to be constant.
4. The measured thermocouple temperatures and the material properties are the boundary condition and input to the software, respectively.

##### Algorithm

***Step 1:*** Primarily, the software considers the known heat flux as the boundary condition and then it estimates all the nodal temperatures by solving a 2D transient heat conduction equation (Eq. 16) stated below.

16 $$\rho \,{C}_{p}\,\frac{\partial T}{\partial t}=k\left[\frac{{\partial }^{2}T}{\partial {x}^{2}}+\frac{{\partial }^{2}T}{\partial {y}^{2}}\right]$$

***Step 2:*** A nonlinear optimization technique (Crank-Nicolson formulation) was used to perform the final prediction of temperature distribution from the assumed surface heat fluxes^23^. The above technique decreases the error between the predicted and measured thermocouple temperatures at the same location.

***Step 3:*** When the minimum error is less than 0.02 °C, the above process converges. If it is more than the converged value, then INTEMP again estimates the new surface heat flux and iterates until it reaches the lowest error.

***Step 4:*** At the converged heat fluxes, the correct temperatures are determined.

#### Calculation of various parameters

1. Heat transfer coefficient (*h*)
  In the current work, the heat transfer coefficient has been calculated from the surface heat flux (*Q*) and driving force values.
2. Developed condition ($$\frac{\dot{{\boldsymbol{m}}}\,{{\boldsymbol{C}}}_{{\boldsymbol{p}}}}{{\boldsymbol{h}}\,{{\boldsymbol{A}}}_{{\boldsymbol{s}}}}$$)

The value for the developed condition is determined from heat transfer coefficient (*h*), mass flow rate ($\dot{m}$), specific heat capacity (${C}_{p}$) and contact area (*A*)_*s*_.

#### Error analysis

In the current study, the quenching experiments such as low, medium, high and air atomized spray cooling were considered. All the experiments were conducted thrice and the average values were reported. The errors stated in the manuscript are the deviation from the average values. The maximum and minimum values of errors obtained for the calculation, prediction or measurements of various parameters are illustrated in Table 1.

**Table 1:** Errors associated in the current work.

Sl no.	Name of the parameters	Type of error	Minimum error	Maximum error
1	Surface heat flux (MW/m^2^)	Predicted	0.002	0.005
2	Surface temperature (°C)	Predicted	5	8
3	Water flow rate (m^3^/s)	Measured	0.15 × 10^−5^	0.22 × 10^−5^
4	Airflow rate (Normal m^3^/h)	Measured	0.3	0.5
5	Heat transfer coefficient (W/m^2^ °C)	Calculated	6	8
6	Impingement density (kg/m^2^s)	Calculated	3	5
7	Residence time (ms)	Calculated	0.002	0.005

As the reported surface heat flux and heat transfer coefficient curves contain huge data points in the temperature ranging from 900 to 30 °C, therefore, the errors in form of error bars have not been incorporated in Fig. 5 to avoid the clumsiness of the figures. Before experimentation, the measuring sensors were calibrated. The precautions suggested in the literature for the minimum error in the output of the experimentation were considered^24^.

## Data Availability

The data generated during the experiments will be available on request to the corresponding author.
